# HSF1 is involved in immunotherapeutic response through regulating APOJ/STAT3-mediated PD-L1 expression in hepatocellular carcinoma

**DOI:** 10.1080/15384047.2022.2156242

**Published:** 2022-12-08

**Authors:** Hongxia Cheng, Sikai Wang, Aidan Huang, Jing Ma, Dongmei Gao, Miaomiao Li, Huaping Chen, Kun Guo

**Affiliations:** a Liver Cancer Institute, Zhongshan Hospital, Fudan University, Key Laboratory of Carcinogenesis and Cancer Invasion Ministry of Education, Shanghai, People’s Republic of China; b Guangxi Zhuang Autonomous Region Women and Children Care Hospital, Guangxi, China; c Department of Clinical Laboratory, First Affiliated Hospital of Guangxi Medical University Nanning, Guangxi, China

**Keywords:** Hepatocellular carcinoma, immunotherapy, HSF1, PD-L1, APOJ, JAK/STAT3

## Abstract

Hepatocellular cancer (HCC) is a serious illness with high prevalence and mortality throughout the whole world. For advanced HCC, immunotherapy is somewhat impactful and encouraging. Nevertheless, a substantial proportion of patients with advanced HCC are still unable to achieve a durable response, owing to heterogeneity from clonal variability and differential expression of the PD-1/PD-L1 axis. Recently, heat shock factor 1 (HSF1) is recognized as an important component of tumor immunotherapeutic response as well as related to PD-L1 expression in cancer. However, the mechanism of HSF1 regulating PD-L1 in cancer, especially in HCC, is still not fully clear. In this study, we observed the significantly positive correlation between HSF1 expression and PD-L1 expression in HCC samples; meanwhile combination expressions of HSF1 and PD-L1 served as the signature for predicting prognosis of patients with HCC. Mechanistically, HSF1 upregulated PD-L1 expression by inducing APOJ expression and activating STAT3 signaling pathway in HCC. In addition, we explored further the potential values of targeting the HSF1-APOJ-STAT3 axis against CD8^+^ T cells-mediated cancer cells cytotoxicity. These findings unveiled the important involvement of HSF1 in regulating PD-L1 expression in HCC as well as provided a novel invention component for improving the clinical response rate and efficacy of PD-1/PD-L1 blockade.

## Introduction

Immune checkpoint inhibitors (ICI) including anti-cytotoxic T lymphocyte-associated protein 4 (anti-CTLA-4), anti-programmed cell death 1 (PD-1) and anti-PD-L1 antibodies have gained approval for the treatment of a variety of cancers in these years.^1^ Recent progresses in advanced HCC demonstrate the immunotherapy with ICIs is effective and promising. Whereas, there is still a considerable proportion of patients with advanced HCC who cannot achieve durable response,^2^ mainly due to the great intra-cancer heterogeneity from clonal diversity^3,4^ and different expression of PD-1/PD-L1 signaling axis among individual patients.^5^ Upregulated PD-L1 often contributes to cancer immune evasion and finally leads to cancer progress.^6,7^ Hence, understanding comprehensively the association between cancer heterogeneity and immune checkpoint expressions and making the corresponding therapeutic strategies will be critical for improving the efficacy and achieving the precise immunotherapy.

As we know, hepatocellular carcinoma (HCC) is one of the most heterogeneous cancers. Hence, it is impossible to accurately capture the emerging node of cancer heterogeneity; in contrast, it is more practical to seize the “vulnerability” of cancer.^8^ In almost all cancer cells, molecular chaperone proteins tend to be highly expressed, for stabilizing the conformation of mutated oncogenic proteins and contributing to cell survival and proliferation. This phenomenon is known as “molecular chaperone addiction”, which is being recognized as the vulnerability against cancer.^9^ As the most important molecular chaperone, heat shock proteins (HSPs) do not only maintain the homeostasis of mutated proteins and enhance the malignant phenotype of cancer, but also act as a “capacitor” for phenotypic diversity and finally contribute to the accumulation of intracellular genetic variation and accelerate the immune escape mediated by clonal evolution of cancer cells.^10^

Heat shock factor 1 (HSF1) is a specific transcriptional factor which can orchestrate the expression of HSPs and protect cancer cells from microenvironmental and drug-induced stresses.^11^ In the past few years, HSF1 has been demonstrated to enhance the immune response and anti-cancer efficacy,^12^ for example through increasing the natural killer cell-activating ligands^13^ and manipulating the TCR signaling network.^14^ Of note, it has been recently reported that HSF1 activation correlated with ICI response in cancer patients^15^ and there is a positive association between HSF1 and PD-L1 expression in cancer tissues.^16^ However, the mechanism of HSF1 regulating PD-L1 in cancer, especially in HCC, is still not fully clear.

APOJ, also known as clusterin, is a chaperone and found to be involved in a variety of biological events including tumor progression, cell death and neurodegenerative or cardiovascular diseases through orchestrating the chaperone-client complex.^17,18^ In these years, APOJ has been demonstrated to be closely related to immune response numerous inflammatory and immune responses,^19,20^ particularly in liver diseases.^21^ Notably, APOJ is recently recognized as one prominent extracellular chaperone in modulating inflammation, extracellular matrix remodeling, proteostasis and signaling transduction in the tumor microenvironment.^22^ As a critical signaling transductor, STAT3 functions also as an important transcription factor and participates in the regulation of various immune cells and their crosstalk with TME,^23^ suggesting to targeting STAT3 signaling may be a promising option in the tumor immunological treatment field, along with the emergence of new STAT3 inhibitors:^24^ in practical, the targeting blockage of STAT3 signaling has shown an exciting efficacy in HBV-related HCC.^25^

In this current study, we report that HSF1 upregulated PD-L1 expression by inducing APOJ expression and activating STAT3 signaling pathway in HCC, which will provide an experimental evidence for the possibility of making a precise strategy against cancer heterogeneity as well as improving immunotherapy efficacy in HCC.

## Results

### The association between HSF1 expression and PD-L1 expression in HCC

**Figure 1.**
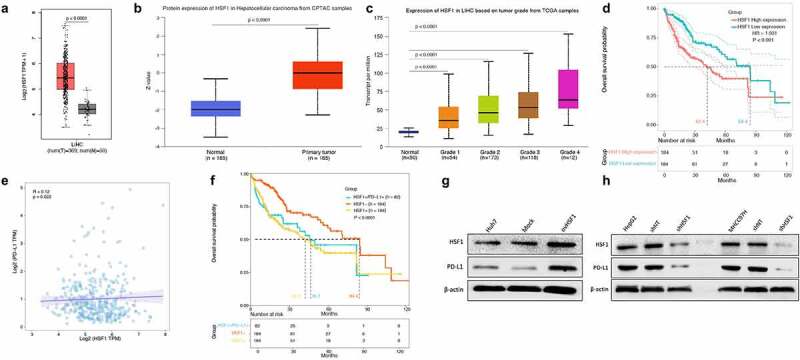
A positive association between HSF1 expression and PD-L1 expression in HCC including cancer tissues and cell lines, showing HSF1 downregulation leading to the downregulation of PD-L1 expression.

Here, we performed firstly a bioinformatics analysis on HSF1 expression feature in HCC by exploring the public cancer databases including GEPIA, UALCAN and Oncomine databases. It was found that HSF1 expression was significantly increased in HCC, compared to normal tissue (Figure 1a-b); the increased expression of HSF1 was associated with cancer stages in HCC (Figure 1c). Moreover, poor prognosis was found in HCC patients with high-expressed HSF1 (Figure 1d). In these years, some studies demonstrated HSF1 is involved in cancer-specific immune response.^12^ Herein, the association between HSF1 expression and immune-related signatures was further explored. It was shown that HSF1 expression displayed a positive correlation with some cancer immune microenvironment-related cell populations including a variety of T cell signatures and cancer-associated fibroblasts, but no significant correlation with B cells and microphage (**Supplementary Fig.1**).

It is well-known that cancer cells evade T cell immune attack through expressing highly PD-L1.^26^ Therefore, the correlation analysis was performed to explore whether HSF1 expression is associated with PD-L1 expression in liver cancer. The result showed that there was a positive correlation between HSF1 expression and PD-L1 expression in liver cancer (Figure 1e). Unexpectedly and notably, for patients with high HSF1 expression, when PD-L1 was simultaneously highly expressed, the prognosis of these patients was not obviously improved (figure 1f). To clarify the reason why PD-L1 presented different expression pattern among HSF1 positive liver cancer, we observed firstly the expression pattern of HSF1 and PD-L1 in a variety of HCC cell lines including Huh7, PLC, HepG2, MHCC97H, Hep3B, SNU354, SNU449 and JHH1 and found that the baseline expression was low for both HSF1 and PD-L1 in Huh7 cells; in contrast, the other HCC cell lines with high HSF1 harbored high levels of PD-L1 (**Supplementary Fig.2)**. To further determine the correlation between HSF1 expression and PD-L1 expression in HCC, we observed the effect of HSF1 expression alteration on PD-L1 expression in HCC cells. The result showed that PD-L1 was decreased after HSF1 knockdown in HepG2 and MHCC97H with high baseline HSF1 levels; meanwhile, PD-L1 was increased after HSF1 overexpression in Huh7 with low baseline HSF1 (Figure 1g-h).

### HSF1-induced PD-L1 expression requires the involvement of APOJ

To explore the mechanism of HSF1-induced PD-L1 expression in HCC, we extracted genes positively related with HSF1 and PD-L1 respectively as well as negatively related with HSF1 in LIHC from UALCAN database (http://ualcan.path.uab.edu) and further analyzed through performing a GO/KEGG analysis. Of note, it was also interestingly found that both for HSF1 and for PD-L1, the positive related genes were obviously enriched in the common biological process-protein binding; meanwhile genes negatively related with HSF1 were dominantly enriched in the regulation of complement activation (**Supplementary Fig.3**), suggesting the involvement of a potential candidate which cannot only be responsible for upregulated chaperon activity, but also contribute to downregulated complement activation in the process of HSF1-induced PD-L1 expression in HCC.

**Figure 2.**
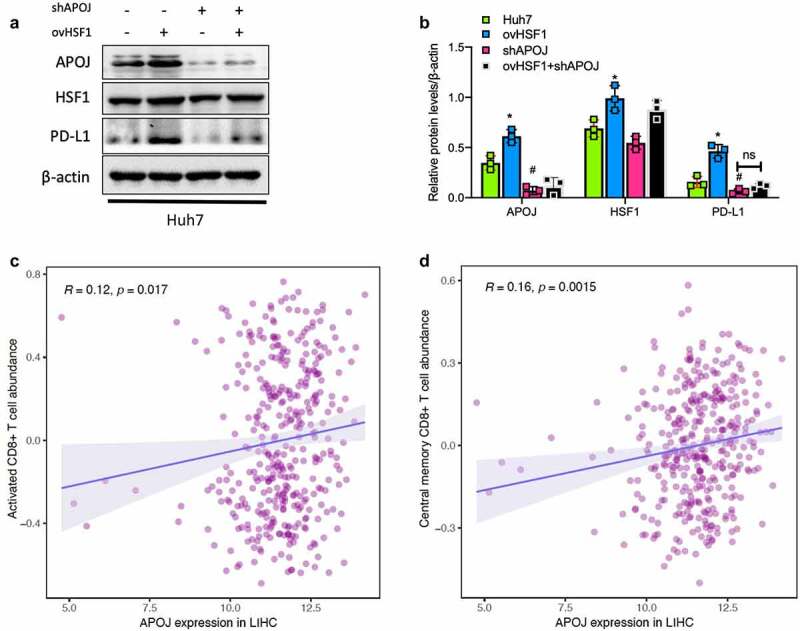
The regulation of HSF1 on PD-L1 expression requires the involvement of CD8 + T signature-related APOJ, showing a positive correlation between APOJ expression and abundance of CD8 + T cells in HCC.

Recently, a chaperone, APOJ (as clusterin), has been suggested to be involved in several basic biological events such as the maintenance of protein homeostasis imbalance-induced cell death, cancer progression, and neurodegenerative disorders;^27,28^ meanwhile it was verified to serve as a complement cascade inhibitor in a variety of conditions.^29^ Hence, to investigate whether APOJ is involved in the effect of HSF1 on PD-L1 expression, we performed a APOJ knockdown followed by HSF1 overexpression in HCC cells and found that after APOJ knockdown, HSF1 overexpression did not significantly upregulate the expression of PD-L1 in HCC cells (Figure 2a-b). The result suggested that HSF1-induced PD-L1 expression mentioned above depended on the existence of APOJ in HCC cells.

### STAT3 signaling as a downstream of APOJ contributes to HSF1-induced PD-L1 expression in HCC

Based on the finding mentioned above, a further analysis on the correlation between APOJ expression and immune cells infiltration was performed and the result showed that APOJ expression was significantly correlated with CD8 + T cells infiltration in HCC (Figure 2c-d). In addition, the analysis on the correlation between APOJ expression and CD8 + T cells in pan-cancers showed that APOJ expression correlated with CD8 + T signature particularly with effector T cells (**Supplementary Fig.4A**). Analysis on the molecular functions of genes positively correlated with PD-L1 showed these genes were dominantly involved in SH2 domain binding, MHC complex binding and kinase activity, suggesting the regulation of kinases with SH2 domain possibly responsible for the PD-L1 expression (**Supplementary Fig.4B**). These results suggested a potential association between APOJ and PD-L1 expression through a signaling harboring SH2 domain.

**Figure 3.**
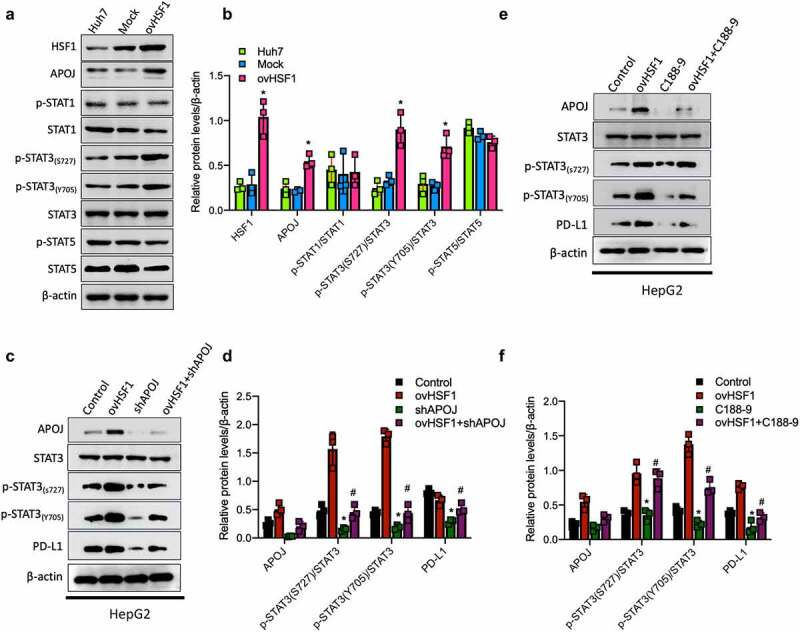
STAT3 signaling as a downstream of APOJ contributes to HSF1-induced PD-L1 expression in HCC, reflecting by the effects of HSF1 or APOJ on the levels of p-STAT3/STAT3.

It is recently recognized that JAK-STAT signaling is closely associated with immune evasion and resistance to immune checkpoint inhibitors in HCC^30,31^ as well as the function of STAT protein family depends on SH2 domain and play a crucial role in immune homeostasis.^32^ Herein, we firstly observed the activation of JAK-STAT signaling in the condition of HSF1 expression alteration in HCC cells. The results showed that, after HSF1 overexpression, the ratios of p-STAT3(S727)/STAT3 and p-STAT3(Y705)/STAT3 were significantly increased while the ratios of p-STAT1/STAT1 and p-STAT5/STAT5 were not significantly changed in Huh7 cells (Figure 3a-b). Subsequently, we detected the alteration of STAT3 activation when upregulating HSF1 expression in HCC cells (HepG2) with APOJ knockdown in order to verify the role of APOJ in modulating the JAK-STAT3 signaling activity. It was shown that APOJ knockdown reduced significantly the ratio of p-STAT3(S727)/STAT3 and p-STAT3(Y705)/STAT3 and PD-L1 expression; however, HSF1 overexpression after APOJ knockdown didn’t increase completely the levels of p-STAT3/STAT3 and PD-L1 expression in HepG2 cells (Figure 3c-d). In addition, we investigated furtherly the change of STAT3 activation and PD-L1 expression by using C188-9, a specific inhibitor of STAT3. It was observed that C188-9 treatment decreased significantly the levels of p-STAT3/STAT3 as well as PD-L1 expression, which was partly rescued when HSF1 overexpression in HepG2 cells (Figure 3e-f).

### *Explored treatments* in vitro *against HSF1-APOJ-STAT3 axis*

**Figure 4.**
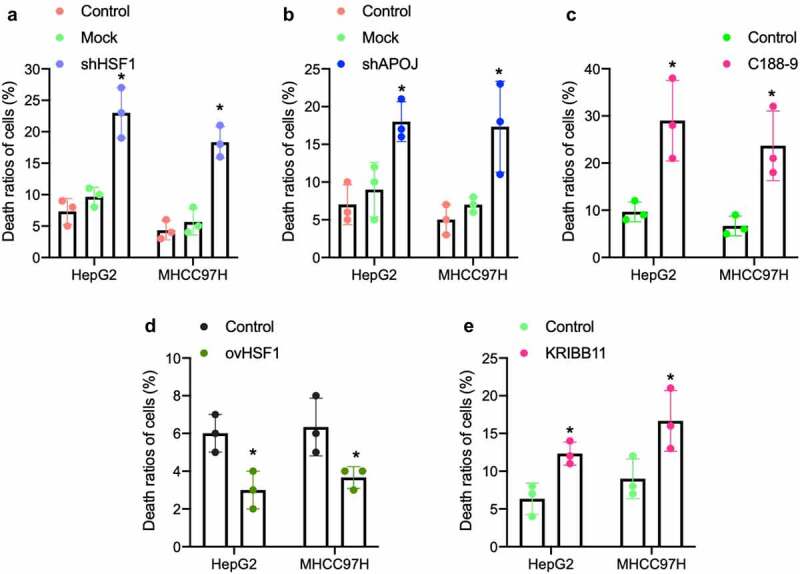
Targeting intervention on HSF1-APOJ-STAT3 axis affects CD8 + T cells-mediated cytotoxicity for HCC cells by using the specific inhibitor *in vitro.*

To define the effect of HSF1-APOJ-STAT3 axis on CD8^+^ T cells-mediated cancer cells cytotoxicity *in vitro*, we co-cultured isolated CD8^+^ T cells with HCC cells and found that in HCC cells with HSF1 knockdown, CD8^+^ T cells-mediated cytotoxicity significantly enhanced (Figure 4a); similar result was also observed in HCC cells with APOJ knockdown and C188-9 treatment (Figure 4b-c). Expectedly, CD8^+^ T cells-mediated cytotoxicity was significantly inhibited in HCC cells with HSF1 overexpression (Figure 4d). In addition, HSF1 inhibitor also sensitized significantly CD8^+^ T cells-mediated cytotoxicity in HCC cells (Figure 4e). All of these results suggested the potential inhibition of HSF1-APOJ-STAT3 axis strengthened CD8^+^ T cells-mediated cytotoxicity through downregulating the expression of PD-L1 in HCC cells.

## Discussion

The application of ICIs in malignant cancers is a key milestone in the immunotherapy for cancer patients.^33^ In recent years, ICIs have made breakthroughs in the systemic treatment of advanced HCC, but there are still many uncertainties in the clinical application, including obviously different efficacy among individuals, which leads to the considerable interest in the regulatory mechanism of PD-L1 contributing to immune escape in HCC.^34,35^ It has been recognized that PD-L1 expression is a key factor affecting immunotherapy in HCC,^36^ therefore clarifying the regulatory mechanism of PD-L1 expression in HCC may provide more evidence when making an immunotherapy or immune combination therapy strategy for HCC. In the study, we observed the significantly positive correlation between HSF1 expression and PD-L1 expression in HCC samples; meanwhile combination expressions of HSF1 and PD-L1 served as the signature for predicting prognosis of patients with HCC. Mechanistically, HSF1 upregulated PD-L1 expression by inducing APOJ expression and activating STAT3 signaling pathway in HCC. In addition, we explored further the potential values of targeting the HSF1-APOJ-STAT3 axis against CD8^+^ T cells-mediated cancer cells cytotoxicity. These findings unveiled the important involvement of HSF1 in regulating PD-L1 expression in HCC as well as provided a novel invention component for improving the clinical response rate and efficacy of PD-1/PD-L1 blockade.

As mentioned above, the phenomenon that only a fraction of cancer patients are sensitive to ICI-based immunotherapy proposes the necessity of investigating the resistance mechanism in specific immunological TME of HCC. Recently, it has been demonstrated that the immunological configuration of TME doesn’t only exhibit some signs of oncogenic pathway-driven immunosuppression (such as PD-L1 upregulation), but also requires stress response pathways to support the establishment of immunological ‘cold’ TME (also as non-oncogene addiction).^37^ The term non-oncogene addiction means these stress response pathways are unable to drive oncogenesis by themselves, but are required for cancer progression through orchestrating TME. As a transcription factor, HSF1 involves its versatile abilities to various stress response pathways via inducing the expressions of heat shock proteins and serves as a potent multifaceted modifier of cancer progression, whereby the involvement of HSF1 in cancer is described as a classic non-oncogene addiction.^38^ Our previous study showed that the non-oncogene HSF1 displayed the robust prognostic and predictive values in HCC.^39^ In the current study, we further found the unfavorable survival of HSF1-positive patients with simultaneous PD-L1 high-expression. It was reported that PD-L1 high expression is correlated with survival and tumor recurrence in HCC patients;^40^ meanwhile PD-L1^+^ HCC displayed an activated immune microenvironment.^41^ Likewise, we observed a positive correlation between HSF1 expression and PD-L1 expression in liver cancer. The result suggested the potentials of HSF1 in immune mechanisms of HCC, possibly related to HSF1 inducing PD-L1 expression in HCC; therefore addressing the issue will help to make a novel therapeutic strategy in HSF1 positive HCC patients.

Moreover, in our previous studies, it has been demonstrated that HSF1 promoted malignant phenotype and drug resistance of HCC through regulating glucose and lipid metabolism and mediating the alteration of metabolic status via cross-talking with immune cells in HCC.^42–45^ These findings indicate a possible role of HSF1 in orchestrating TME of HCC. Recently, more evidences show that HSF1 promotes cancer-specific immune response and is being recognized as a better target for neoadjuvant immunotherapy.^12,46^ Furthermore, as a transcription factor, HSF1 can directly initiate the expression of MHC class I molecules, thereby promoting immune recognition and immunotherapy,^15^ suggesting the regulation of HSF1 on the expression of immune molecules in cancer. Here, we investigated the association between HSF1 and PD-L1 expression in HCC and verified that PD-L1 can be induced by HSF1 in HCC cells based on a series of experiments in vitro. The result suggested the necessity of investigating further the regulatory mechanisms of PD-L1 by HSF1 in HCC.

In liver cancer, PD-L1 is regulated by multiple factors and signaling pathways at multiple levels including epigenetics, transcriptional/post-transcriptional regulation, and post-translational modification.^35^ PD-L1 is often overexpressed on cancer cells, thereby evading immune surveillance; it has also been reported that PD-L1 overexpression can make cancer cells more sensitive to PD-1/PD-L1 inhibitors.^47^ Of late, it is reported that HSF1 can enhance PD-L1 expression by binding to the latter’s promotor in some other types of cancers,^16^ however the mechanism underlying HSF1-induced PD-L1 expression in HCC is still unclear. To clarify the potential factor involved in HSF1-induced PD-L1 expression in HCC, we performed a bioinformatics analysis on genes positively related with HSF1 and PD-L1 respectively as well as negatively related with HSF1 in LIHC extracted from several public cancer databases and found HSF1-induced PD-L1 expression depended on the existence of APOJ in HCC cells. As is well-known, APOJ can serve as a multifunctional molecule involved in inflammation and immunity.^48^ Our previous study identified also APOJ as an inducer of epithelial-mesenchymal transition (EMT) contributing to HCC metastasis;^49^ of note, the current finding represents a novel function of APOJ in HCC, meanwhile extends our understanding of its roles in establishing immune TME.

In addition, based on rational bioinformatics analysis, we surmised a potential kinase with SH2 domain may be involved in the association between APOJ and HSF1-indeuced PD-L1 expression. STAT is a transcription factor containing the SH2 structural domain. Importantly, the SH2 structural domain of STAT is able to specifically bind the phosphorylated tyrosine residues and form STAT dimers, which can translocate into the nucleus and activate target genes that are important for cell proliferation and cell survival.^50^ The important example of STAT signaling contributing to cancer development comes from STAT3. The mutated STAT3 is often constitutively activated and functions as an oncogenic protein.^51^ It has recently been recognized that STAT3 serves as a central node in the regulation of the anti-tumor immune response.^52^ Of note, STAT3 is being implicated as a promising target to anti-cancer immunity in HCC.^30^ In the study, we further observed the activation of STAT3 signaling after HSF1 overexpression and such STAT3 signaling activation was dependent on the existence of APOJ in HCC cells. The result suggested that STAT3 signaling as a downstream of APOJ contributes to HSF1-induced PD-L1 expression in HCC cells. Over the last decade, the mechanistic connection between STAT3 signaling and PD-L1 expression has been indicated in several cancers including liver cancer, bladder cancer and colorectal cancer.^53–55^ We also demonstrated the inhibition of STAT3 signaling can significantly downregulate the expression of PD-L1, which required APOJ existence in HCC cells. The regulation of PD-L1 expression by APOJ-mediated STAT3 signaling in HCC is firstly reported in our study. Taken together, these observations in the current study indicated HSF1 can regulate PD-L1 expression through mediating APOJ and activating STAT3 signaling in HCC cells as well as suggest HSF1-APOJ-STAT3 axis may serve as a potential actionable node to re-construct the immunological status of TME in HCC. Hence, we evaluated furtherly the effects of targeting HSF1-APOJ-STAT3 axis on CD8^+^ T cells-mediated cancer cells cytotoxicity and verified the enhancement of CD8^+^ T cells-mediated cytotoxicity when HSF1-APOJ-STAT3 axis was inhibited in HCC cells. The observation may be explained by the downregulation of PD-L1 expression as proposed and demonstrated in the current study.

There are some limitations in the study. For example, exact roles of APOJ in HSF1-induced PD-L1 expression needs to be further investigated and the associated molecular mechanism needs to be explored. Moreover, whether pharmacological or genetic inactivation or activation of HSF1-APOJ-STAT3 axis can inflame the TME of HCC in vivo? Nevertheless, we explored a potential crosstalk between oncogenic signaling and stress response signaling in orchestrating immune TME of HCC and suggest a novel mechanism of HSF1-induced PD-L1 expression; moreover, based on the finding that HSF1 induced PD-L1 by regulating STAT3 signaling in HCC, a combination of HSF1 inhibitor or STAT3 inhibitor and PD-1 antibodies would be expected as more effective therapeutic approaches to target against TME in HCC, even pan-cancer.

## Materials and Methods

### Bioinformatics analysis

GEPIA (http://gepia.cancer-pku.cn/) analysis, based on the UCSC Xena project and The Cancer Genome Atlas (TCGA), contains RNA sequencing expression data for cancer and normal tissues. In this study, the mRNA expression level of HSF1 in adjacent normal tissues and liver hepatocellular carcinoma (LIHC) samples was analyzed. Additionally, we further analyzed the correlation between HSF1 and PD-L1 mRNA expression levels in LIHC samples via utilizing the database. Moreover, in our study, the mRNA expression and protein expression levels of HSF1 and clinical data in normal and LIHC samples were analyzed through utilizing UALCAN (http://ualcan.path.uab.edu) and Oncomine (https://www.oncomine.org/) analysis, based on The Cancer Genome Atlas (TCGA) and Clinical Proteomic Cancer Analysis Consortium (CPTAC), cancer-related microarray and high-throughput sequencing, contains RNA sequencing expression, protein expression and clinical data for cancer and normal samples. In addition, we also downloaded genes positively associated with HSF1 and PD-L1 respectively as well as and negatively associated with HSF1 in LIHC samples from the database for Gene Ontology (GO) and the Kyoto Encyclopedia of Genes and Genomes (KEGG) analysis. After matching the ensemble gene IDs to gene symbols according to the annotation data (gencode.v22.annotation.gene.probeMap) downloaded from UCSC Xena website (http://xena.ucsc.edu/), the gene expression values of TCGA-LIHC samples were transformed into transcripts per million value (TPM) and fetched the logarithm on per million value respectively. According to the median value of HSF1 and PD-L1 mRNA expression, we set HSF1-high expression (HSF1+), HSF1-low expression (HSF1-) and PD-L1-high expression based on HSF1-high expression (HSF1+/PD-L1+) three groups. The mRNA expression of three groups, cancer grade and survival data in TCGA-LIHC samples were chosen for performing Pearson correlation and Kaplan-Meier survival analyses on the basis of the previous online analyses.

GO and KEGG enrichment analysis of HSF1-related genes and PD-L1-related genes downloaded from UALCAN database was performed using cluster Profiler R package to explore biological characteristics. Overall survival curves based on Kaplan–Meier survival analysis were performed between three groups mentioned above by utilizing survival and survminer R package. Log-rank test, Gehan-Breslow-Wilcoxon test and Cox proportional-hazards model with three groups verified by GraphPad Prism 9.0 software were carried out in our study. The correlation analysis between HSF1 and PD-L1 mRNA expression levels in TCGA-LIHC samples was implemented via using ggpmisc R package in our study. Visualization of all analyses mentioned above was mapped by using ggplot2 R package. All revalidation of online analyses mentioned previously and visualizations in TCGA-LIHC samples were performed in the R programming language (Version 4.1.3) using multiple R packages.

## Cell culture

The human HCC cell lines (Huh7, PLC, HepG2, MHCC97H, Hep3B, SNU354, SNU449, and JHH1) were obtained from the Liver Cancer Institute at Zhongshan Hospital, and they were cultured in Dulbecco’s modified Eagle’s medium (DMEM) (Yuanpei, China) supplemented with 10% fetal bovine serum (FBS) (Biosharp, China). However, SNU354 and SNU449 were grown in RPMI medium 1640 (Yuanpei, China), which was supplemented with 10% fetal bovine serum (FBS) (Biosharp, China) and 1% penicillin-streptomycin (GIBCO, Canada). Cells were grown at 37°C in a humidified environment of 5% CO2 and were treated according to the experiment design. The Ethics Committee of Zhongshan Hospital, Fudan University, approved the use of the cell lines.

## Generation of lentivirus-mediated HSF1 or APOJ knockdown and overexpression

HSF1 overexpression, HSF1 knockdown, and APOJ knockdown plasmids and the control plasmids were created by Jikai Co (Shanghai, China). The envelope plasmid, packaging plasmid, and shRNA-expressing plasmid were co-transfected into 293 T cells to create the lentiviruses. 48–72 hours after cells were transfected, virus-containing medium was collected. Following incubation with the lentivirus-containing medium, the cells were then treated with puromycin for constructing the stable-transfection cells.

### Cell transfection and inhibitor treatment

The indicated cells were planted in 6-well plates for transient transfection experiments. Opti-MEM Reduced Serum Medium (Thermo Fisher Scientific, USA) was used to transfect the plasmids into cells according to the manufacture’s protocols. After the appropriate timepoints, one portion of the cell was treated with C188-91(10 mM), a small-molecule selective inhibitor of STAT3, and knockdown efficacy was determined using Western blotting.

### Western blotting

The Western blot was carried out as previously reported.^43^ In brief, RIPA lysate (Biyuntian Company, China) was being used to lyse the cells seeded in 6-well plates. The protein concentration was measured by using BCA method after the harvest was centrifuged at 15,000 g for 15 min at 4°C. Electrophoresis of a total of 20 µg equivalent protein per loading well in 10% sodium dodecyl sulfate (SDS)-polyacrylamide gel was conducted. The proteins were then separated and transferred to polyvinylidene difluoride (PVDF) membranes (Millipore Corporation, USA). These membranes were incubated overnight at 4°C with specific antibody (see Supplementary table 1) at a dilution of 1:1000 in the primary antibody dilution (Beyotime Company, China), and were detected with HRP-conjugated anti-rabbit IgG. The development of the films was performed by using the enhanced chemiluminescence detection system (Perkin-Elmer Life Sciences, USA). The bands were quantified densitometrically using Quantity One software (Bio-Rad Laboratories, USA).

### CD8^+^ T cell-mediated cytotoxicity

CD8^+^T cells were obtained from peripheral blood mononuclear cells (PBMC) by using the Naïve CD8^+^ T cell isolation kit (#130-093-244, Nuowei Biotech Co., Beijing) combination with flow cytometric sorting. Then, CD8^+^ T cells were added and co-cultured with HCC cells at the ratio of 12:1 in the 96-well plate. After CD8^+^ T cells in the supernatant were washed out by PBS buffer, the remaining HCC cells was collected and detected by using CCK-8 kit (Dojindo, Japan). The calculated death proportion to stand for the CD8^+^ T cell-mediated cytotoxicity ability.

### CCK-8 assay

The cells in 96-well plates were added with CCK8 solution (100 uL of DMEM media with 10 uL of CCK8) and incubated for additional 1 h at 37°C using the CCK8 (Dojindo, Japan) according to the manufacturer’s instructions. Then, while calculating cell viability, the absorbance readings were read at a wavelength of 450 nm. Each sample was examined repeatedly three times.

### Statistical analysis

All revalidation of online analyses mentioned previously and visualizations in TCGA-LIHC samples were performed in the R programming language (Version 4.1.3) using multiple R packages. All the statistical and computational analyses were implemented via utilizing R programming language or GraphPad Prism 9.0 software. 95% confidence intervals (CIs), Hazard ratios (HRs) and median overall survival time were calculated in our Cox proportional- hazards model. Pearson’s correlation coefficient was conducted to determine the correlation between HSF1 and PD-L1 mRNA expression levels in TCGA-LIHC samples. The Kaplan–Meier method was used to compare the survival rates between three groups. All the data came from at least three separate in vitro trials. All data were reported as means ± SD and were judged statistically significant at P < .05. For group comparisons, the student’s t-test was utilized. All statistical tests were conducted bilaterally. with p < .05 being statistically significant. *, p < .05; **, p < .01; ***, p < .001; ****, p < .0001; NS, not significant.

## Supplementary Material

Supplemental Material

## Data Availability

All the data generated or analyzed during this study are included in this article.
