# Supplementary material for: HSF1 is involved in immunotherapeutic response through regulating APOJ/STAT3-mediated PD-L1 expression in hepatocellular carcinoma
Source: Cancer Biol Ther. 2022 Dec 8;24(1):1–9. doi: 10.1080/15384047.2022.2156242 (PMC9746510; doi:10.1080/15384047.2022.2156242)
Supplement: Supplemental Material [file KCBT_A_2156242_SM1688.zip › Alternative Text for all figures.docx]

**Main figures**

**Figure 1.** A positive association between HSF1 expression and PD-L1 expression in HCC including cancer tissues and cell lines, showing HSF1 downregulation leading to the downregulation of PD-L1 expression.

**Figure 2．**The regulation of HSF1 on PD-L1 expression requires the involvement of CD8+T signature-related APOJ, showing a positive correlation between APOJ expression and abundance of CD8+T cells in HCC.

**Figure 3.** STAT3 signaling as a downstream of APOJ contributes to HSF1-induced PD-L1 expression in HCC, reflecting by the effects of HSF1 or APOJ on the levels of p-STAT3/STAT3.

**Figure 4.** Targeting intervention on HSF1-APOJ-STAT3 axis affects CD8+ T cells-mediated cytotoxicity for HCC cells by using the specific inhibitor *in vitro*.

**Supplementary Figures**

**Supplementary Figure 1**. The pattern about the association between HSF1 expression and immune-related signatures including Naïve T cells, effector T cells, memory T cells, exhausted T cells in TCGA-LIHC samples.

**Supplementary Figure 2**. HSF1 shows a positive association with PD-L1 in a variety of HCC cell lines including Huh7, PLC, HepG2, MHCC97H, Hep3B, SNU354, SNU449 and JHH1.

**Supplementary Figure 3**. The bioinformatic analysis showing the potential association between HSF1 and PD-L1; the two molecules share some mutual molecular functions and biological processes.

**Supplementary Figure 4**. A strong correlation between APOJ and abundance of 28 tumor-infiltrating lymphocytes across 30 human cancers; meanwhile enrichment of PD-L1-positively correlated genes into 30 molecular functions.
