# Supplementary material for: HSF1 is involved in immunotherapeutic response through regulating APOJ/STAT3-mediated PD-L1 expression in hepatocellular carcinoma
Source: Cancer Biol Ther. 2022 Dec 8;24(1):1–9. doi: 10.1080/15384047.2022.2156242 (PMC9746510; doi:10.1080/15384047.2022.2156242)
Supplement: Supplemental Material [file KCBT_A_2156242_SM1688.zip › supplemental files-revision.docx]

**Supplementary materials**

**Supplementary Figure Legends**

**
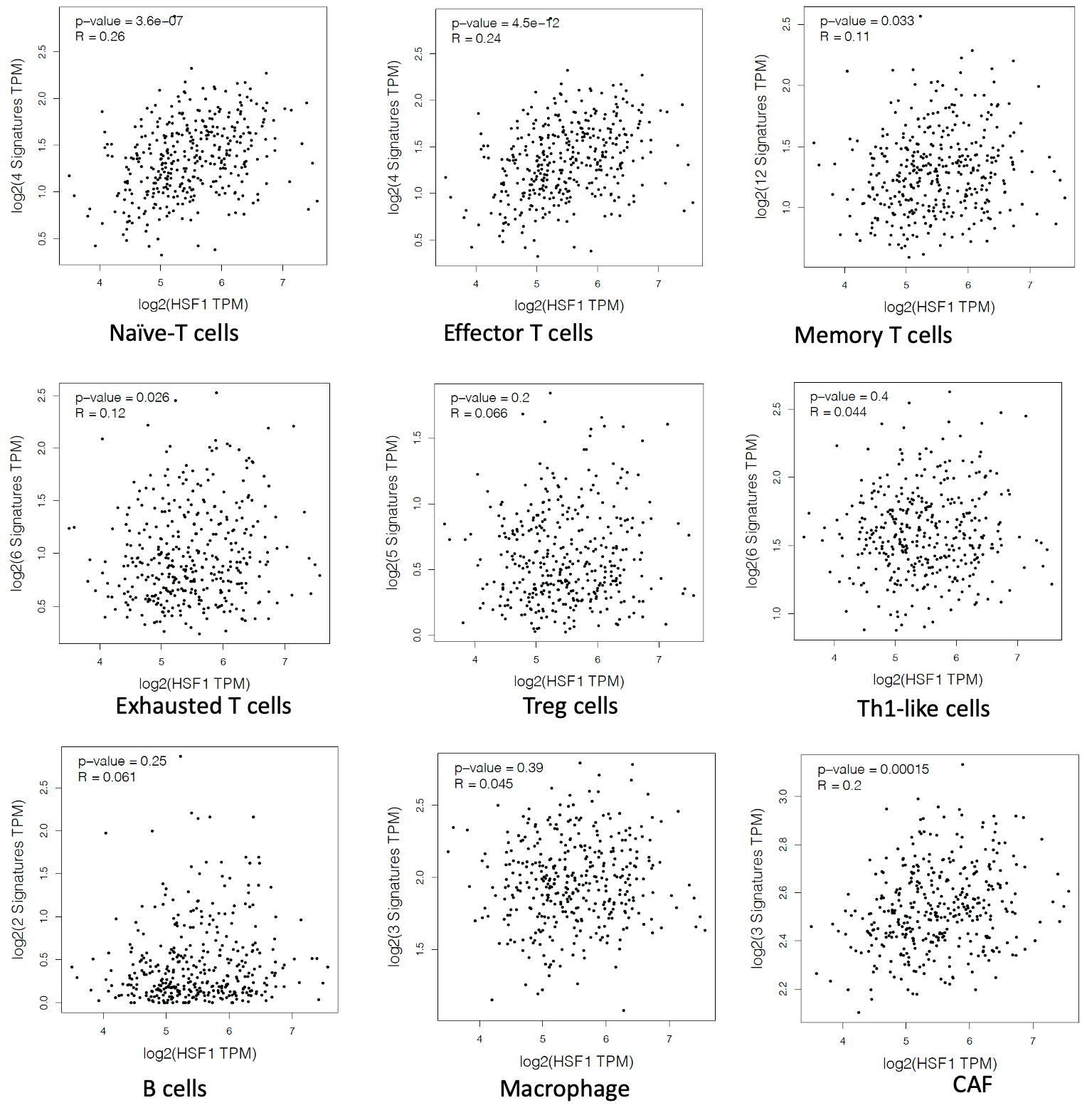
**

**Supplementary Figure 1. Analysis on the association between HSF1 expression and immune-related signatures in liver cancer.** Pearson's correlation coefficient was conducted to determine the correlation between HSF1 and Naïve T cells, effector T cells, memory T cells, exhausted T cells, Treg cells, Th1-like cells, B cells, macrophage and CAF in TCGA-LIHC samples.

**
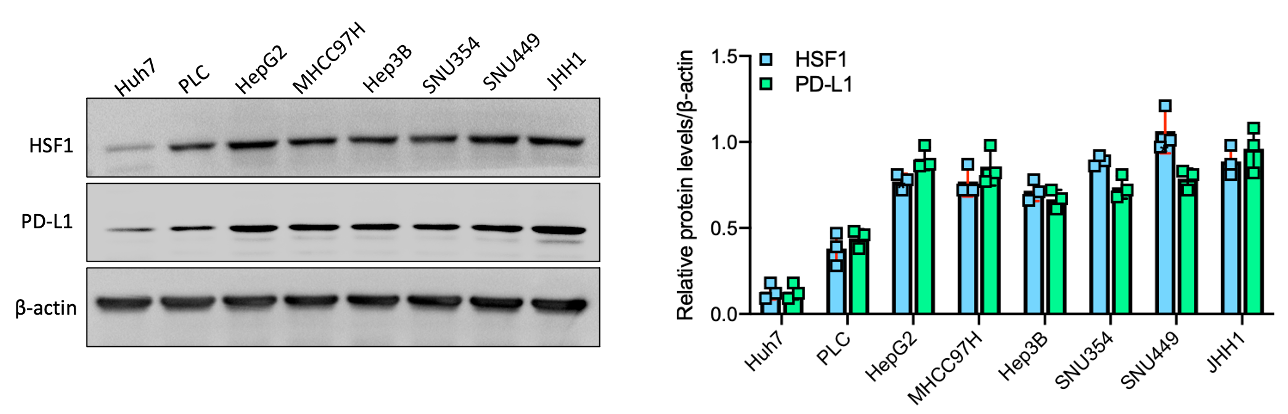
**

**Supplementary Figure 2. The favorable association between HSF1 and PD-L1.** HSF1 and PD-L1 protein expression levels in 8 HCC cells including Huh7, PLC, HepG2, MHCC97H, Hep3B, SNU354, SNU449 and JHH1 were detected using Western blot. Data are presented as mean standard deviation. Three separate experiments were carried out.


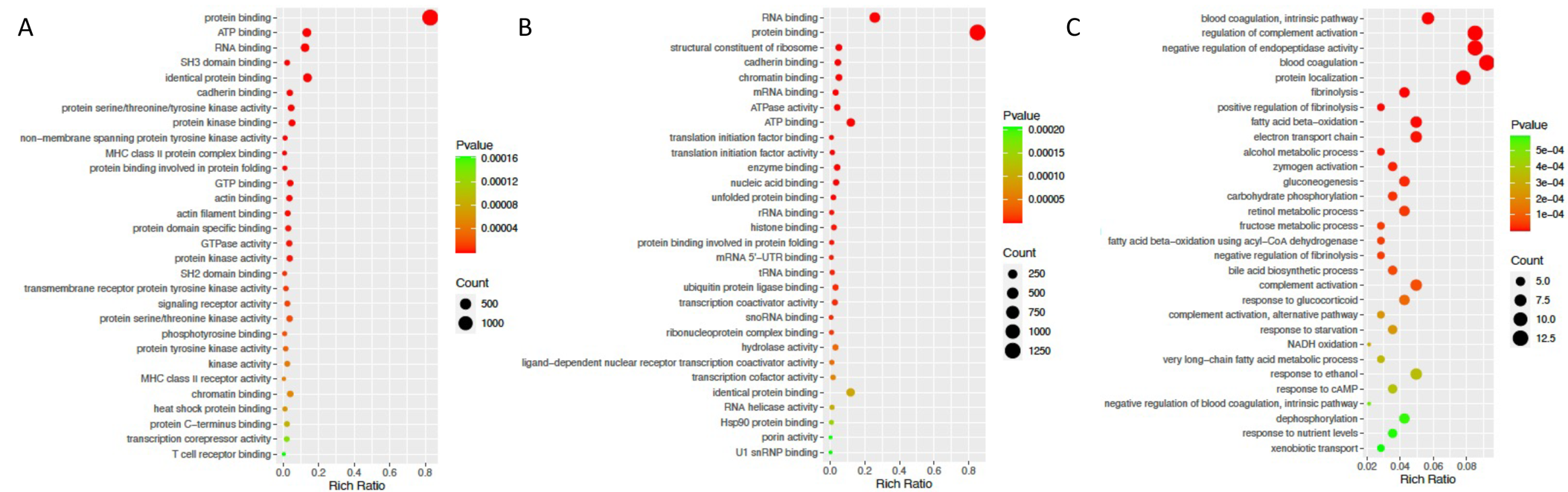


**Supplementary Figure 3. The bioinformatic analysis on the potential association between HSF1 and PD-L1.** (**A-B**) Top 30 molecular function (MF) terms of Gene ontology (GO) enrichment analysis with genes positively correlated with PD-L1 and HSF1, respectively. All GO-MF terms were ordered by P value. (**C**) Top 30 biological process (BP) terms of Gene ontology (GO) enrichment analysis with HSF1-negatively correlated genes. All GO-BP terms were ordered by P value.

**
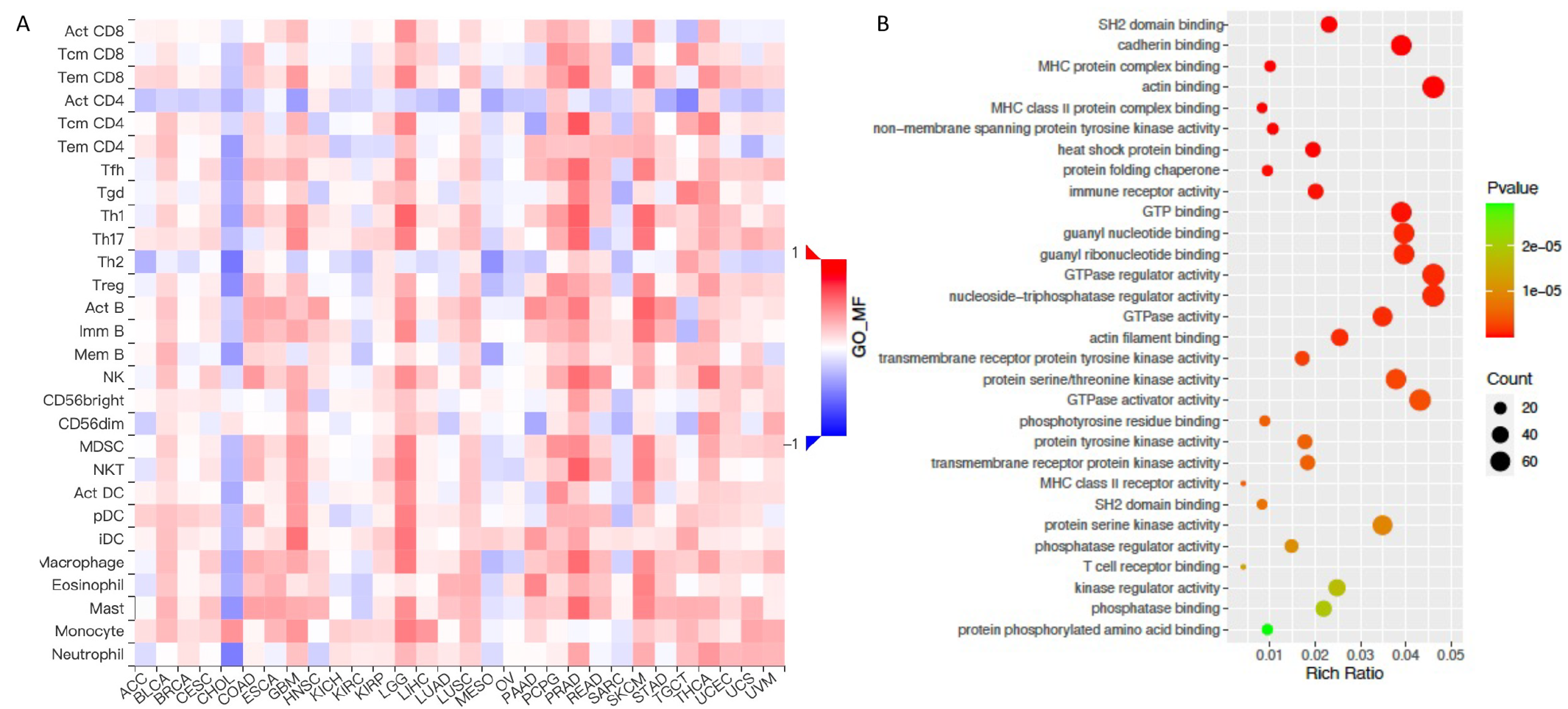
**

**Supplementary Figure 4.** (**A**) Spearman’s correlation analysis between APOJ mRNA expression level and abundance of 28 tumor-infiltrating lymphocytes (TILs) across 30 human cancers in TISIDB database. The legend showed the correlation coefficient ranged from plus one to minus one. The gradient red section represented the positive correlation between APOJ mRNA expression and the abundance of the TIL. The gradient blue section represented the negative correlation between APOJ mRNA expression and the abundance of the TIL. ACC, adrenocortical carcinoma; BLCA, bladder urothelial carcinoma; BRCA, breast invasive carcinoma; CESC, cervical squamous cell carcinoma and endocervical adenocarcinoma; CHOL, cholangiocarcinoma; COAD, colon adenocarcinoma; DLBC, lymphoid neoplasm diffuse large B‐cell lymphoma; ESCA, esophageal carcinoma; GBM, glioblastoma multiforme; HNSC, head and neck squamous cell carcinoma; KICH, kidney chromophobe; KIRC, kidney renal clear cell carcinoma; KIRP, kidney renal papillary cell carcinoma; LAML, acute myeloid leukemia; LGG, brain lower grade glioma; LIHC, liver hepatocellular carcinoma; LUAD, lung adenocarcinoma; LUSC, lung squamous cell carcinoma; MESO, mesothelioma; OV, ovarian serous cystadenocarcinoma; PAAD, pancreatic adenocarcinoma; PCPG, pheochromocytoma and paraganglioma; PRAD, prostate adenocarcinoma; READ, rectum adenocarcinoma; SARC, sarcoma; SKCM, skin cutaneous melanoma; STAD, stomach adenocarcinoma; TGCT, testicular germ cell tumors; THCA, thyroid carcinoma; THYM, thymoma; UCEC, uterine corpus endometrial carcinoma; UCS, uterine carcinosarcoma; UVM, uveal melanoma. (**B**) Top 30 molecular function (MF) terms of Gene ontology (GO) enrichment analysis with PD-L1-positively correlated genes. All GO-MF terms were ordered by P value.

**Table S1. All antibodies information and sources in Western blot in this study.**

| MARKER (SPECIES) | DILUTION | DISTRIBUTOR/SOURCE  (CATALOGUE NUMBER) |
| --- | --- | --- |
| Primary antibodies: |  |  |
| HSF1 Rabbit mAb | 1:1000 | # 12972S |
| clusterin Rabbit mAb | 1:1000 | # 42143S |
| STAT1 Rabbit mAb | 1:1000 | #14994T |
| P-STAT1 Rabbit mAb | 1:5000 | #9167S |
| STAT3 Rabbit mAb | 1:1000 | # 12640S |
| P-STAT3(S727) Rabbit mAb | 1:1000 | #34911T |
| P-STAT3(Y705) Rabbit mAb | 1:2000 | #9145S |
| STAT5 Rabbit mAb | 1:1000 | # 25656ST |
| P-STAT5 Rabbit mAb | 1:1000 | #4322T |
| PD-L1 Rabbit Antibody | 1:1000 | #13684T |
| Secondary antibodies: |  |  |
| Anti-rabbit IgG HRP-linked Ab | 1:5000 | #5127S |
|  |  |  |
