# Supplementary figures and images for: HSF1 is involved in immunotherapeutic response through regulating APOJ/STAT3-mediated PD-L1 expression in hepatocellular carcinoma
Source: Cancer Biol Ther. 2022 Dec 8;24(1):1–9. doi: 10.1080/15384047.2022.2156242 (PMC9746510; doi:10.1080/15384047.2022.2156242)

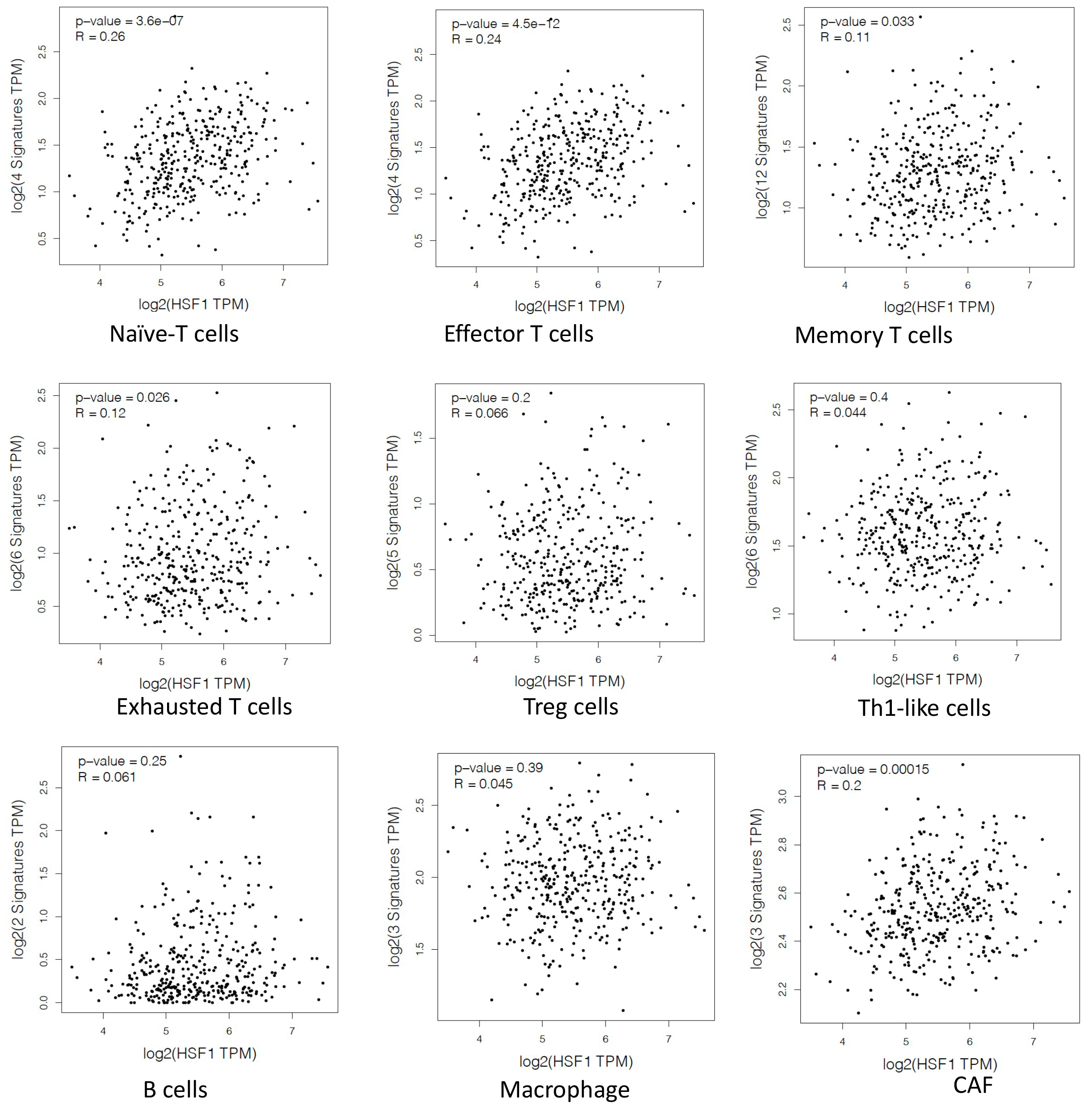

Supplement: Supplemental Material [file KCBT_A_2156242_SM1688.zip › sFig1.tif]

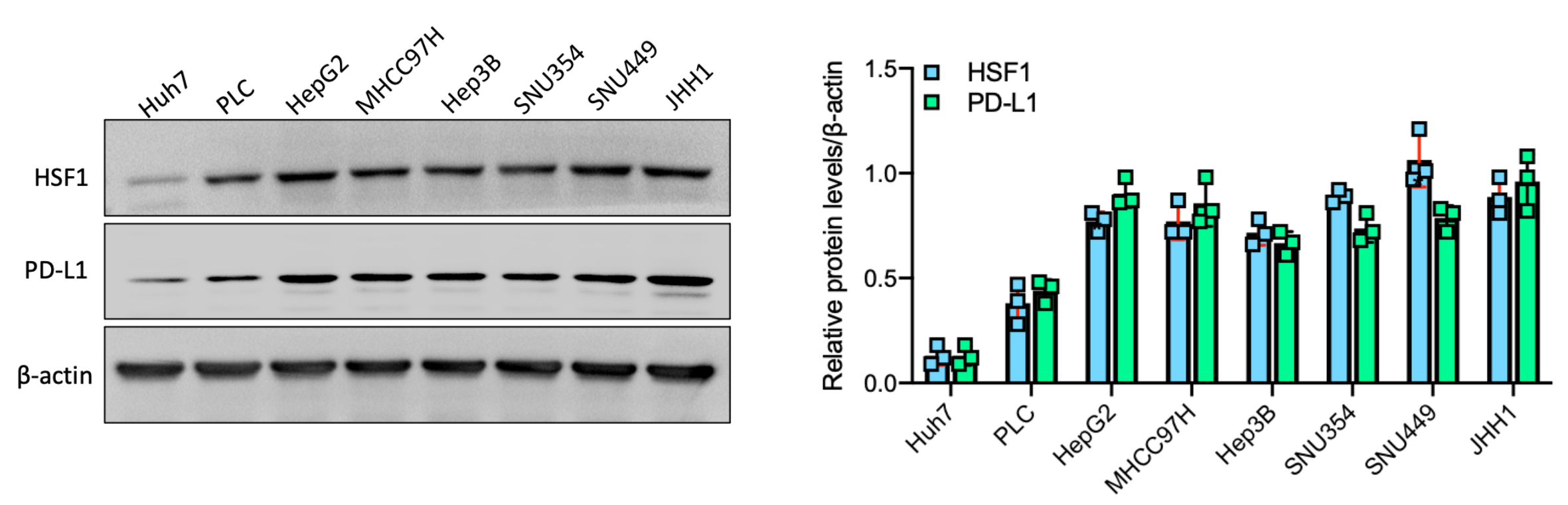

Supplement: Supplemental Material [file KCBT_A_2156242_SM1688.zip › sFig2.tif]

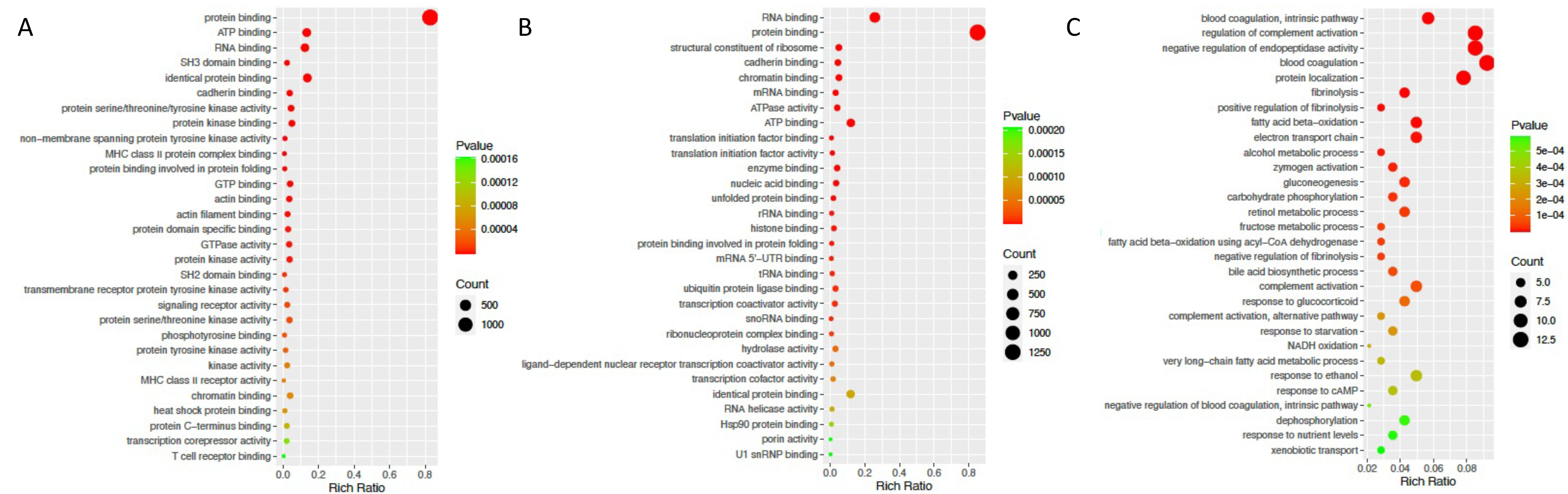

Supplement: Supplemental Material [file KCBT_A_2156242_SM1688.zip › sFig3.tif]

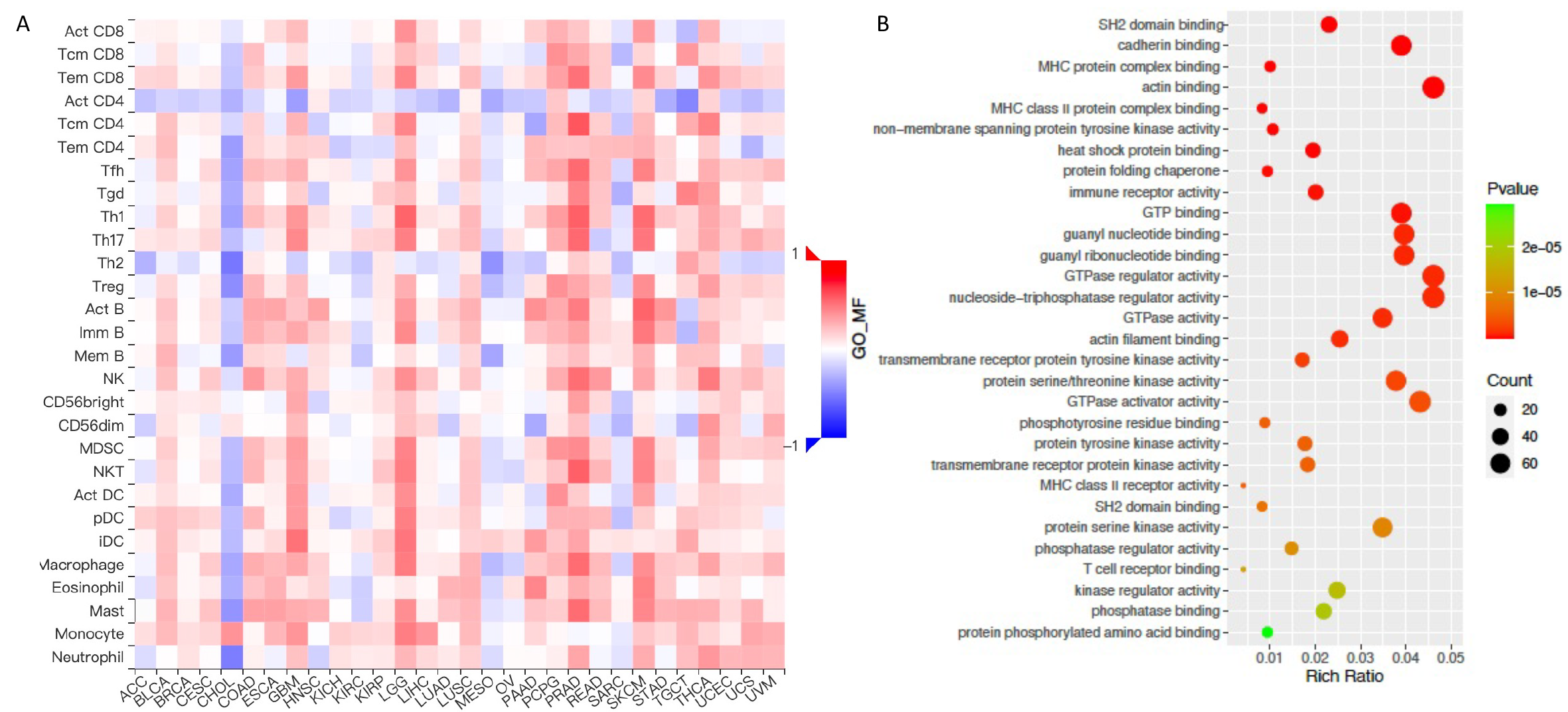

Supplement: Supplemental Material [file KCBT_A_2156242_SM1688.zip › sFig4.tif]
